# Individuality in nutritional preferences: a multi-level approach in field crickets

**DOI:** 10.1038/srep29071

**Published:** 2016-06-30

**Authors:** Chang S. Han, Heidi Y. Jäger, Niels J. Dingemanse

**Affiliations:** 1 Behavioural Ecology, Department of Biology, Ludwig-Maximilians University of Munich, Planegg-Martinsried, Germany

## Abstract

Selection may favour individuals of the same population to differ consistently in nutritional preference, for example, because optimal diets covary with morphology or personality. We provided Southern field crickets (*Gryllus bimaculatus)* with two synthetic food sources (carbohydrates and proteins) and quantified repeatedly how much of each macronutrient was consumed by each individual. We then quantified (i) whether individuals were repeatable in carbohydrate and protein intake rate, (ii) whether an individual’s average daily intake of carbohydrates was correlated with its average daily intake of protein, and (iii) whether short-term changes in intake of carbohydrates coincided with changes in intake of protein within individuals. Intake rates were individually repeatable for both macronutrients. However, individuals differed in their relative daily intake of carbohydrates versus proteins (i.e., ‘nutritional preference’). By contrast, total consumption varied plastically as a function of body weight within individuals. Body weight—but not personality (i.e., aggression, exploration behaviour)—positively predicted nutritional preference at the individual level as large crickets repeatedly consumed a higher carbohydrate to protein ratio compared to small ones. Our finding of level-specific associations between the consumption of distinct nutritional components demonstrates the merit of applying multivariate and multi-level viewpoints to the study of nutritional preference.

Individuals from the same population often vary in their relative consumption of different prey or macronutrients^1^,^2^,^3^. Individual animals typically utilize only a restricted part of the nutritional range available to the population, either by choice or necessity^1^. Indeed, prey preference has been demonstrated to covary with digestive ability^4^, morphology^5^, and level of intraspecific competition^6^,^7^. While phenotypic variation in diet characterizes most natural populations^8^,^9^, and populations are known to differ in diet preference^1^,^10^,^11^, little is known about whether individuals of the same population differ consistently in their preference for nutritional components (e.g. macronutrients)^12^. For example, are individuals repeatable in total (‘intake rate’) or relative consumption (‘nutritional preference’ or ‘intake target’) of different macronutrients when repeatedly making such choices?

Nutritional preference may be usefully viewed as a labile phenotypic character that can vary both within and among individuals^13^,^14^. This notion is implied by meta-analyses demonstrating that behavioural traits generally harbour repeatable (also called ‘among-individual’) variation^15^ while simultaneously varying plastically within individuals^16^. ‘Among-individual variation’ represents the variation in behavior in the population that is caused by differences in average behavior among repeatedly assayed individuals, while ‘within-individual variation’ captures the extent of variation in behaviour that is observed across observations of a single individual. Repeatability, in turn, is the proportion of total variation (i.e., among plus within individuals) that is due to differences among individuals.

Repeatable variation is commonly called ‘animal personality’ in the behavioural ecology literature^17^,^18^, and has particularly been studied in the context of behaviours that facilitate resource acquisition at the cost of increased likelihood of disease, predation or parasitism^19^,^20^,^21^,^22^,^23^. A prominent idea in this literature is that personality types might represent the behavioural mechanism by which trade-offs between life-history traits^24^ are mediated, where proactive (aggressive, bold, explorative or reproductively active) types are predicted to adopt a relatively fast pace-of-life compared to more reactive individuals^17^. Individual differences in pace-of-life have been suggested to also affect nutrient intake and diet preference, for example, because proactive individuals overexpress energetically costly activities^25^,^26^. Proactive males are predicted to be more active, more aggressive, and to bias investment towards current reproduction (e.g., by courting females), and are therefore predicted to prefer carbohydrate-rich diets^14^. In addition, given the common effect of nutritional intake on body weight^27^, individual differences in nutrient intake are also predicted to relate positively to body weight. The existence of nonzero repeatability in nutritional intake rate or preference has, however, received little attention to date^14^ though work on fruit flies suggests that intake rate is heritable^28^ thus logically repeatable^29^.

In this paper, we describe an experiment that quantified an individual’s consumption of two key macronutrients (carbohydrates and proteins) presented repeatedly as two synthetic food sources to >80 adult males of the Southern field cricket (*Gryllus bimaculatus*) of a single population (Tuscany, Italy). Our aim was to test whether individuals of the same population differed in intake rate and target. Male crickets are known to prefer carbohydrate-rich diets (with an approximate 5.7:1 ratio of carbohydrates to protein; Han & Dingemanse, in prep, see also ref. 30), however, whether individuals are repeatable in nutritional preferences has not been explored. We therefore quantified (i) whether individuals were repeatable in intake rate (i.e., the total amount of each macronutrient consumed) and nutritional preference, and (ii) whether an individual’s average daily intake rate of carbohydrates was correlated with its average daily intake of protein (causing a so-called ‘among-individual correlation’), and (iii) whether short-term changes in intake rate of carbohydrates coincided with changes in intake of protein within individuals (causing a so-called ‘within-individual correlation’)^31^,^32^. We expected tight positive within-individual correlations, provided that total nutrient consumption varied plastically within individuals. We also expected tight positive among-individual correlations, provided that individuals did not differ in nutritional preference. We further quantified whether intake rates (of carbohydrates and proteins) and nutritional preference were correlated with known proxies of proactivity (aggressiveness, exploration), mating activity, and body weight among- and within-individuals.

## Results

Carbohydrate intake and protein intake were both individually repeatable (Carbohydrate intake: R = 0.25, SE = 0.06; Protein intake: R = 0.24, SE = 0.05; Table S1). The angle (in radians) between the carbohydrate axis and the rail (a line connecting the origin (0, 0) and the individual intake target, Fig. S1) for individual nutritional preference derived by arctangent-transforming carbohydrate:protein ratios^33^,^34^ was also individually repeatable (R = 0.23, SE = 0.06; Table S1). This implied that individuals were also repeatable in the ratio of carbohydrates vs. protein that they consumed.

**Figure 1:**
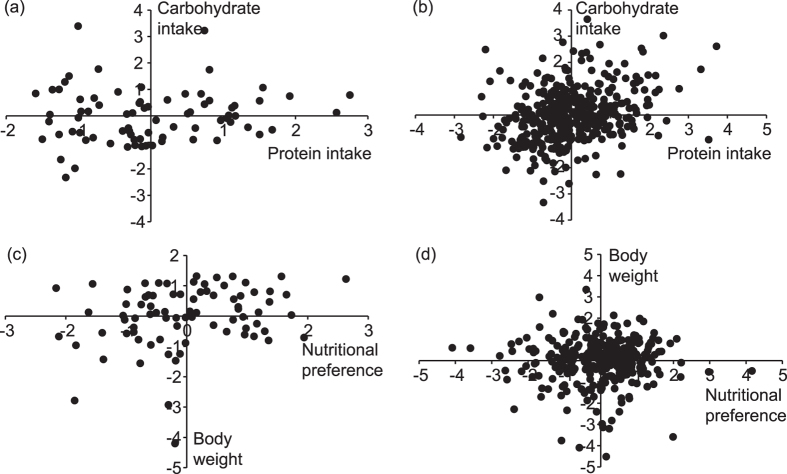
(**a**) The among-individual correlation (n = 82 individual means) and (**b**) the within-individual correlation (n = 383 observations) between carbohydrate and protein intake, and (**c**) the among-individual correlation (n = 82 individual means) and (**d**) the within-individual correlation (n = 383 observations) between body weight and nutritional preference in Southern field crickets. Following^31^, the plot of the among-individual correlation is visualized as the correlation between means of each individual’s z-transformed phenotypes; the within-individual correlation is visualized as the correlation between the z-transformed deviations of each observation from a focal individual’s mean for both phenotypes. Larger values of nutritional preference indicate that individuals prefer more carbohydrates over proteins.

When applying an alternative variance-partitioning approach to test for individual differences in nutritional preference, we found that the amount of carbohydrate intake was positively correlated with the amount of protein intake within individuals (*r*_*W*_ = 0.30, SE = 0.05, χ^2^_1_ = 29.0, P < 0.001, Table 1, Fig. 1a), whereas the among-individual correlation between average carbohydrate and protein intake did not significantly deviate from zero (*r*_*A*_ = −0.04, SE = 0.20, χ^2^_1_ = 0.1, P = 0.75, Table 1, Fig. 1b). In other words, when individuals up-regulated their consumption of carbohydrates from one day to the next, they also up-regulated their consumption of protein (*r*_*W*_ > 0), while an individual’s average consumption of carbohydrates was not associated with its average consumption of protein (*r*_*A*_ ~ 0). Importantly, the among-individual correlation deviated significantly from one (χ^2^_0.5_ = 35.2, P < 0.001); this confirmed our finding that intake rates of carbohydrates vs. protein did not represent the same trait, i.e., that individuals were repeatable nutritional preference (i.e., the carbohydrate:protein ratio that they consumed).

Neither intake rates of carbohydrates or protein nor nutritional preference were correlated with any of the behavioural traits, whether within or among individuals (Table 1). Body weight and nutritional preference were correlated positively among though not within individuals (Table 1, Fig. 1bc). In other words, large animals repeatedly consumed relatively more carbohydrates compared to proteins but this nutritional preference did not shift with body weight within individuals among days. These level-specific relationships between weight and nutritional preferences emerged because body weight was significantly positively correlated with carbohydrate (but not with protein) intake among-individuals, while body weight was significantly positively correlated with both carbohydrate and protein intake within-individuals (Table 1).

## Discussion

This study demonstrates that nutrient intake rate represents a repeatable individual characteristic within an insect population. This is in line with the evidence that nutrient intake rate is heritable^28^. Importantly, the degree of the arctangent-transformed carbohydrate:protein ratio was also individually repeatable, which implies the existence of repeatable individual differences in nutritional preference. This notion of individual-specific intake targets was additionally supported by a variance-partitioning approach, where we examined within- and among-individual correlations between consumption of the two macronutrients (carbohydrate and protein). During days where a male consumed carbohydrate relatively more compared to its own average consumption, it would also consume relatively more protein, thereby causing a tight positive within-individual correlation (Table 1; Fig. 1b). However, the among-individual correlation between the two macronutrient intakes was close to zero and differed significantly from one; this finding implies that our crickets consumed different proportions of the two macronutrients (Table 1; Fig. 1a). Taken together, crickets were plastic in total intake rate across days but simultaneously repeatable in their preference for macro-nutritional composition.

Throughout the animal kingdom, individuals within the same population differ in prey preference and resource use^1^. Previous research on vertebrates has convincingly demonstrated within-species (and among-population) variation in diet and prey consumption by showing individual variation in isotope variation in body tissues^35^,^36^ or gut content^37^. However, only few researchers have repeatedly assayed an individual’s intake behaviour to determine individuality (i.e., repeatability) in prey preference (reviewed in ref. 3). While we firmly demonstrate individual repeatability in feeding specialization, our results further imply that individuals substantially differed in nutritional preference when repeatedly subjected to a nutrient choice experiment. Individuals thus choose to utilize a narrow nutritional niche to meet their specific physiological demands. A recent follow-up experiment, using an independent dataset, confirmed our finding of individual repeatability in feeding specialization in this species (Han & Dingemanse, In Progress), implying that our result is reproducible across datasets of the same species. A key outstanding question is whether this form of individuality represents a ubiquitous characteristic of animal populations^38^, whether it is proximately mediated by developmental plasticity, experience, or genes, and what ecological factors moult this variation.

Body weight represented an individual-level predictor of nutritional preference. Heavier individuals more strongly preferred carbohydrate over protein. This finding is in accordance with the notion that larger animals require more fuel to power energetically costly activities^39^. This interpretation was also warranted by the positive among-individual correlation between weight and carbohydrate intake, and the absence of an among-individual correlation between weight and protein intake. By contrast, repeatable differences in intake were not associated with exploration, aggression, or mating activity, which unexpectedly implies that personality types did not differ in intake rate or nutritional preference. Further research should investigate whether the hypothesized link between nutritional preference and personality is population- or species-specific depending on ecological factors such as availability and diversity of nutrients^40^. Furthermore, when an individual up-regulated its body weight, it also up-regulated its carbohydrate and protein intake, leading to positive within-individual correlations between the three traits; this finding implies that the crickets were subject to day-to-day variation in nutritional demands (i.e., induced by unmeasured micro-environmental variation such as temperature fluctuations).

One key outstanding topic for future research is whether selection acts on nutritional preference. A recent study showed reduced variance in fitness in animal populations forced on a mixed diet compared to one forced on a single-food diet^40^. This finding is essentially corroborated by our study as we show the presence of individual-specific nutritional preferences. When multiple macronutrients are abundant and available in the environment, different types of individual might all meet their specific needs (i.e., individual-specific nutritional preferences) and thus maximise their fitness. In contrast, when a certain macronutrient is deficient in the diet, or few types of food are available in the environment, some individuals might not be able to reach their nutritional preference and thus be disfavoured by selection. In other words, a general implication of our work is that future research should now address whether selection on nutritional preference varies spatiotemporally (as demonstrated recently for other key behaviour traits^41^), and whether environmental heterogeneity in nutritional environments, whether spatial or temporal in nature, can help explain the maintenance of individual variation in feeding specialization observed in the current study.

An important finding of our study is that correlations between intake of different nutrients varied across hierarchical levels (here within- and among-individuals). This implies that they were shaped by different biological mechanisms^24^,^42^,^43^. Individual-specific physiological demands (i.e., caused by variation in size) may underpin individuality in nutritional preference, whereas day-to-day variation in total nutritional demand within individuals caused the amount of both nutritional components to be up- or down-regulated in parallel. Those level-specific mechanisms thus create discordant orientations of correlations across levels, as recently demonstrated also for correlations between cognitive, colour, and personality traits^44^,^45^,^46^. This study thereby highlights the usefulness of joining a character-state perspective with statistical variance-partitioning approaches when testing for the existence of 1) individual specialization in nutritional niches and 2) the role of multiple biological mechanisms in shaping correlations between nutritional components (see also ref. 31). Our finding of level-specific relationships implies that level-specific proximate mechanisms warrant consideration to fully understand the biology of nutritional intake in wild animal populations.

## Methods

### Diet preparation and rearing conditions

We used offspring from a third generation of field crickets *G. bimaculatus* collected from Tuscany (Italy) in July 2014. We selected 82 freshly emerged males from our stock population. Males were subsequently maintained alone in transparent plastic containers (23 × 15 × 17 cm) with an egg carton for shelter and supplied with water and artificial diets *ad libitum*. Two different types of artificial diet, protein-high and carbohydrate-high, each consisting of 60% nutrient content were made according to an established protocol detailed in ref. 47. The protein-high diet contained protein and carbohydrate in a 1:29 ratio (carbohydrate:protein), while the carbohydrate-high diet contained protein and carbohydrate in a ratio of 29:1 (carbohydrate: protein). Proteins consisted of a 3:1:1 mixture of casein, albumen, and peptone, and carbohydrates was made of a 1:1 mixture of sucrose and dextrin. All artificial diets contained Wesson’s salts (2.5%), ascorbic acid (0.275%), cholesterol (0.55%) and a vitamin mix (0.18%), to provide all essential nutrients.

After males had been accustomed to consuming these artificial foods for two weeks, we measured 1) carbohydrate intake and 2) protein intake of each individual by weighing both of the two food dishes every 3 days; we collected 6 repeated measures per individual, i.e. the procedure spanned a period of 18 days. Based on published power analyses^31^, this study design (i.e., combination of number of individuals and number of repeats per individual) yielded high statistical power (~0.9) to detect low levels of repeatability (~0.1). On each of these six sampling occasions, we also performed behavioural assays (exploration, aggression and mating activity) and measured body weight (see below). The artificial foods and water vials were replaced once every 3 days. Males were maintained at 26 °C with 60% relative humidity under a 14L:10D photoperiod.

### Behavioural assay and body weight measure

Exploration, aggression and mating activity were measured in a fixed order at 1–3 minutes intervals, and each set of behavioural assays was conducted every 3 days. All the males were returned to their individual rearing container when a set of behavioural assays was over. Prior to the set of behavioural assays, each male was identified by a small dot of paint (Testors enamel paint) on its pronotum. All the behavioural assays were recorded with a digital camcorder and analysed with tracking software, Noldus Ethovision XT 10 (Noldus Information Technology).

#### Exploration

Two males were removed from their individual containers and placed in a plastic arena with a removable partition in the middle to separate two small rooms (15 × 15 × 10 cm). The two males were separated by an opaque partition in the middle of the arena during the exploration assay. In each compartment, fine-grained white sand was spread on the bottom, and a plastic semi-cylinder was provided as a shelter. The tracking software then measured each male’s total distance moved in the compartment for 10 minutes.

#### Aggression

Aggression assays began when the shelters and the partition in the middle of the arena were removed directly following the exploration assay. Two males then interacted and showed aggression ranging from low-level (e.g. antennal fencing, threat postures) to high-level (e.g. aggressive song stridulation, flaring mandibles and biting). Each aggression trial lasted for 5 minutes. The focal and opponent identities were randomly assigned after the experiment. The tracking software then measured the chasing duration when the focal male chased the opponent to attack (detailed in ref. 48).

#### Mating activity

Following the aggression assay, we gently separated two males with the partition, and then put a female into the arena where the focal male was located. A male and a female then recognized each other and were acclimatized in the arena for 30 seconds. We then measured the average distance between the body centre of a male and the body centre of a female to represent a proxy of male mating activity for 10 minutes (600 secs).

#### Body weight

We weighed each male to the nearest 0.001 g at the end of each behavioural assay. Before returning the male to his home container, we also measured the consumption rates of carbohydrate and protein.

### Statistical analyses

Among- and within-individual correlations between phenotypes (*r*_*A*_ & *r*_*W*_), and repeatabilities (R) of intake rate of each nutrient, nutritional preference (arctangent-transformed carbohydrate:protein ratios^33^,^34^), body weight and behavioural traits, were estimated by fitting z-transformed phenotypic values as multiple response variables into a multivariate mixed-effect model (following procedures detailed in ref. 31). In the model, we included mean-centred sequence (test day) as a fixed effect covariate and random intercepts for individual identity. We assessed the significance of fixed effects using Wald F-tests, and the significance of random effects using likelihood ratio tests (LRTs). The test statistic associated with the LRT was calculated as twice the difference in log likelihood between models with vs. without the focal random effect. To test the effect of individual identity on the phenotype (i.e. repeatability), the value of P was calculated using a mixture of P(χ^2^, df = 0) and P(χ^2^, df = 1) because variances cannot be negative^49^,^50^,^51^. In addition, to test whether the among-individual correlation differed from 1 or 0, we used a CORGH variance structure in ASReml and built models where the among-individual correlation was fixed to one or zero but variances allowed to vary freely (constrained model). First, to test whether correlation deviated from 0, we compared a fully unconstrained model with a model where the among-individual correlation was constrained to 0, and applied a LRT where we tested the observed chi-square against P(χ^2^, df = 1). Next, to test whether a correlation differed from 1, we compared a fully unconstrained model with a model where we constrained the correlation to 1, and applied a LRT where we tested the observed chi-square against a mixture of P(χ^2^, df = 0) and P(χ^2^, df = 1)^49^,^50^,^51^. All analyses were implemented in ASReml 4.0 and solved by restricted maximum likelihood.

## Additional Information

**How to cite this article**: Han, C. S. *et al.* Individuality in nutritional preferences: a multi-level approach in field crickets. *Sci. Rep.* **6**, 29071; doi: 10.1038/srep29071 (2016).

## Supplementary Material

Supplementary Information

## Figures and Tables

**Table 1:** Within- and among-individual correlations between behavioural (exploration, aggression, mating activity) and morphological (body weight) traits, nutrient intake (carbohydrate (C) and protein (P) intake) and nutritional preference (arctangent-transformed C:P ratio).

	Exploration	Aggression	Mating activity	Weight	P intake	C intake
(A) within-individual correlations						
Aggression	−0.09 (0.06)					
Mating Activity	−0.04 (0.06)	0.002 (0.06)				
Weight	−0.11 (0.06)	−0.07 (0.06)	0.01 (0.06)			
P intake	−0.01 (0.06)	−0.06 (0.06)	−0.05 (0.06)	**0.16 (0.06)**		
C intake	0.04 (0.06)	−0.07 (0.06)	0.02 (0.06)	**0.28 (0.05)**	**0.30 (0.05)**	
Nutritional preference	0.06 (0.06)	−0.005 (0.06)	0.06 (0.06)	−0.02 (0.06)	**−0.35 (0.05)**	**0.33 (0.05)**
(B) among-individual correlations						
Aggression	0.05 (0.22)					
Mating Activity	−0.02 (0.21)	−0.34 (0.20)				
Weight	**−0.31 (0.15)**	0.25 (0.15)	−0.15 (0.15)			
P intake	−0.22 (0.20)	0.04 (0.21)	0.30 (0.20)	0.22 (0.15)		
C intake	−0.22 (0.20)	0.33 (0.20)	0.01 (0.20)	**0.68 (0.10)**	−0.04 (0.20)	
Nutritional preference	−0.12 (0.21)	0.03 (0.21)	−0.10 (0.20)	**0.38 (0.14)**	−0.10 (0.51)	**0.20 (0.06)**

(A) Within- and (B) among-individual correlations are provided with standard errors in parentheses. Significant correlations (P < 0.05) are printed in bold-face.
