# Supplementary material for: Individuality in nutritional preferences: a multi-level approach in field crickets
Source: Sci Rep. 2016 Jun 30;6:29071. doi: 10.1038/srep29071 (PMC4928176; doi:10.1038/srep29071)
Supplement: Supplementary Information [file srep29071-s1.pdf]

Individuality in nutritional preferences: a multi-level approach in field crickets

Chang S. Han[1,2], Heidi Jäger [1] and Niels J. Dingemanse[1]

[1]Behavioural Ecology, Department of Biology, Ludwig-Maximilians University of Munich, Planegg-Martinsried, Germany

[2]Current address: School of Biological Sciences, University of Queensland, St Lucia 4072, Australia

hcspol@gmail.com

heidi_jaeger@yahoo.de

n.dingemanse@lmu.de

Supplementary figure

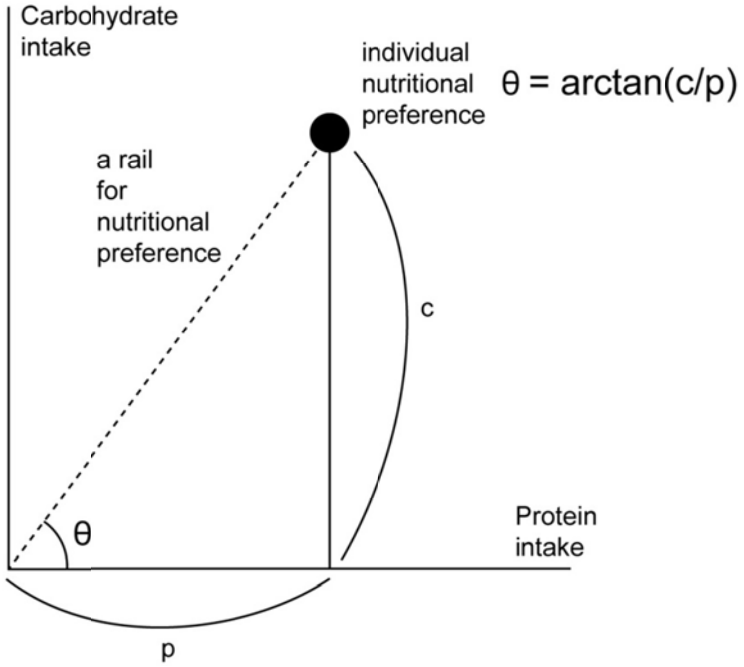

Figure S1. Illustrations of a two dimensional nutrient space indicating an individual's carbohydrate intake, protein intake and nutritional preference. The dotted line from the origin indicates a 'rail' which the individual reaches its nutritional preference. The carbohydrate:protein ratio (c/p) is arctangent-transformed into radians, which represent the individual nutritional preference [1, 2].

Supplementary table

Table S1. Within- and among-individual variances of behavioural (exploration, aggression, mating activity) and morphological (body weight) traits, nutrient intake (carbohydrate (C) and protein (P) intake) and preference (arctangent-transformed C:P ratio). Variance components are provided with standard errors in parentheses.

	Within-individual variance	Among-individual variance
**Exploration**	0.80 (0.07)	0.21 (0.07)
**Aggression**	0.80 (0.07)	0.24 (0.07)
**Mating Activity**	0.79 (0.06)	0.21 (0.06)
**Weight**	0.19 (0.02)	0.86 (0.14)
**P Intake**	0.80 (0.07)	0.24 (0.05)
**C intake**	0.80 (0.07)	0.25 (0.06)
**Nutritional preference**	0.76 (0.07)	0.23 (0.06)

References

[1] Lee, K.P., Kim, J.-S. & Min, K.-J. 2013 Sexual dimorphism in nutrient intake and life span is mediated by mating in Drosophila melanogaster. *Anim. Behav.* **86**, 987-992.

[2] Raubenheimer, D. & Simpson, S. 2003 Nutrient balancing in grasshoppers: behavioural and physiological correlates of dietary breadth. *J. Exp. Biol.* **206**, 1669-1681.
